# Characterization of Microbial Communities in Wastewater Treatment Plants Containing Heavy Metals Located in Chemical Industrial Zones

**DOI:** 10.3390/ijerph19116529

**Published:** 2022-05-27

**Authors:** Taotao Zeng, Liangqin Wang, Xiaoling Zhang, Xin Song, Jie Li, Jinhui Yang, Shengbing Chen, Jie Zhang

**Affiliations:** 1 Hunan Province Key Laboratory of Pollution Control and Resources Reuse Technology, University of South China, Hengyang 421001, China; zengtaotao@usc.edu.cn (T.Z.); wangliangqin00@163.com (L.W.); zhang_xl2021@163.com (X.Z.); sx18873218160@163.com (X.S.); l726827559@163.com (J.L.); yangjinhui@usc.ecu.cn (J.Y.); chenshengbing@usc.edu.cn (S.C.); 2 State Key Laboratory of Urban Water Resources and Environment, Harbin Institute of Technology, Harbin 150090, China

**Keywords:** heavy metals, wastewater treatment, industrial zones, microbial community, human health, water pollution

## Abstract

Water pollution caused by heavy metals (HMs) poses a serious risk to human health and the environment and can increase the risk of diabetes, cancer, and hypertension in particular. In this study, two full-scale wastewater treatment plants (WWTPs) in industrial zones in southern China were selected to analyze the microbial community structure, diversity, similarity, and differentiation in the anoxic/oxic (AO) and anoxic/oxic membrane bioreactor (AO-MBR) units under the stress of HMs. High-throughput sequencing showed that microbial diversity and abundance were higher in the AO process than in the AO-MBR process. In the two WWTPs, the common dominant phyla were *Proteobacteria* and *Bacteroidetes*, while the common dominant genera were *Gemmatimonadaceae*, *Anaerolineaceae*, *Saprospiraceae,* and *Terrimonas*. Manganese (Mn) and zinc (Zn) positively correlated with *Saccharimonadales*, *Nakamurella*, *Micrococcales,* and *Microtrichales*, whereas copper (Cu) and iron (Fe) positively correlated with *Longilinea* and *Ferruginibacter*. Additionally, the relative abundances of *Chloroflexi*, *Patescibacteria*, and *Firmicutes* differed significantly (*p* < 0.05) between the two processes. These results may provide comprehensive outlooks on the characterization of microbial communities in WWTPs, which could also help to reduce the potential environmental risks of the effluent from WWTPs located in industrial zones.

## 1. Introduction

With a growing world population, intensive agriculture, and rapid industrialization, the increase in urban wastewater volumes has proven to be the greatest environmental challenge facing humanity [1]. Moreover, industrial wastewater has led to serious water pollution problems worldwide, since it can contain many harmful chemicals such as acids, base metals, and organic pollutants [2]. Heavy metals (HMs), which include copper, cadmium, zinc, mercury, lead, chromium, iron, nickel, and aluminum in particular, are persistent and toxic and have a wide range of negative health effects [3,4]. Exposure to HMs can lead to various serious human diseases, such as respiratory problems, kidney disease, neurological diseases, and cancer [5]. Previous studies found that arsenic (As) can increase the risk of diabetes mellitus and hypertension, with the threshold level in drinking water of 50 and 150 ppb [6]. Cadmium (Cd) can accumulate in the kidneys when its concentration reaches 1 mg/L and has carcinogenic effects in humans [7]. Thus, wastewater that contains HM should be properly treated before discharge.

In China, industrial wastewater should be pre-treated within an industrial enterprise to meet the quality standards for discharge into municipal sewers (GB/T 31962-2015) [8] before it flows into the WWTPs with municipal wastewater for further treatment. Ultimately, the effluent of WWTPs will reach the discharge standard of pollutants for municipal wastewater treatment plants (GB 18918-2002) [9]. However, there are differences between the two discharge standards, which means that the effluent of industrial enterprise could contain low concentrations of HMs. For example, the maximum permissible effluent concentrations of total Cu and Cd are <2 mg/L and <0.05 mg/L, respectively, according to the wastewater quality standards for the discharge of municipal sewers (GB/T 31962-2015) [8], The discharge standard for municipal WWTPs (GB 18918-2002) is more stringent, and these concentrations should be <0.5 mg/L and <0.01 mg/L, respectively [9]. This means that treating HMs in the wastewater of industrial zones will facilitate both human health and environmental safety.

The activated sludge process is the most widely used biological method in WWTPs worldwide [10]. The low concentration of HMs in the effluent of chemical enterprises could cause risks for functional microorganisms in municipal WWTPs, which affects the sludge properties, extracellular polymers, microbial activity, and microbial community structure. Understanding the microbial structure and function could facilitate sludge population development and improve wastewater treatment efficiency [11,12]. For example, several typical intestinal pathogenic microorganisms might be present in the wastewater, such as *Shigella* spp. or *Salmonella* spp. For instance, *Shigella* spp. infection can cause bacterial dysentery, while *Salmonella* spp. can cause typhoid [13]. Thus, high-throughput sequencing analysis of the microbial community in WWTPs will provide comprehensive information on the microbial population, which will be of benefit for recognition of the pathogenic microorganisms and control of the risk of a pathogenic population in the disinfection unit of WWTPs. Ye et al. (2017) studied the anaerobic/anoxic/aerobic (A^2^O) process to treat mixed wastewater and soy sauce wastewater and found that *Proteobacteria* was the first dominant phylum in the wastewater treatment population [14]. Zeng et al. (2018) investigated the process of treating domestic wastewater in oxidation ditches and found that most bacteria belonged to the *Proteobacteria* and *Bacteroidetes*, while the dominant genera were *Pseudomonas*, *Phycispharea*, and *Methylocystis* [15]. Zhang et al. (2019) used metagenomic sequencing to investigate the functional genes associated with the biotransformation of C, N, P, and S in sequencing batch reactors (SBR) [16]. Furthermore, the presence of functional genes in activated sludge bacteria has been promoted to facilitate the proliferation of oxidizing bacteria and to enhance their oxidation capacity for As(III) oxidation [17].

There have been many investigations into the characteristics of microbial communities in WWTPs. However, few have focused on the chemical industrial zones with WWTPs that contain HMs in their influent [18]. The objective of this study was to determine the structure and diversity of sludge bacterial communities in two different treatment processes by WWTPs in industrial zones. This required an analysis of the types and concentrations of HMs in wastewater, the microbial community structure of sludge in different WWTPs, the effects of treatment processes on bacterial communities, and the gene function prediction of different microorganisms. The present study provides a microbial community characterization of WWTPs in industrial zones, which will help engineers to optimize the wastewater treatment process and reduces the potential harm of effluent to the environment and human health.

## 2. Materials and Methods

### 2.1. Basic Description of the Studied WWTPs

The two WWTPs, referred to as SM-WWTP and XW-WWTP, are both located in two economic and technological development areas in Hunan Province. The SM-WWTP was used to treat mixed wastewater from both industrial enterprises and residential areas, while the XW-WWTP was mainly used to treat industrial wastewater containing HMs. The main process of the SM-WWTP is anaerobic/oxic (AO), with a daily capacity of 200,000 m^3^ of influent. The main process of the XW-WWTP is anaerobic/aerobic, while the membrane bioreactor (AO-MBR) process has a daily treatment capacity of 100,000 m^3^. The designed influent quality of raw sewage in the WWTPs examined is shown in Table 1.

Mixtures of activated sludge and wastewater were collected from the aerobic and anaerobic tanks of the SM-WWTP, designated as SM_A and SM_O, respectively, as well as from the anaerobic, aerobic, and MBR tanks of the XW-WWTPs, denoted as XW_A, XW_O, and XW_M, respectively. The mixtures were precipitated for 30 min without shaking to separate the supernatant from the precipitate and were then stored at −20 °C for the further analyses described in the sections that follow.

### 2.2. DNA Extraction, PCR Amplification, and High-Throughput Sequencing

Total genomic DNA was extracted from the sludge samples using an E.Z.N.A.^®^ Soil DNA Kit (OMEGA, BioTek, Winooski, VT, USA), following the manufacturer’s protocol [19]. DNA quality and quantity were assessed using agarose gel (1%) electrophoresis and spectrophotometry (260 nm/280 nm ratio). The V4 hypervariable regions of the bacterial 16SrRNA gene were amplified using the primer 515FmodF/806RmodR, with a GeneAmp^®^ PCR system 9700 (Applied Biosystems, Waltham, NJ, USA) [17]. High-throughput sequencing was carried out by Shanghai Majorbio Bio-pharm Technology Co., Ltd. (Shanghai, China) on an Illumina MiSeq platform [20]. The sequences with an average quality score greater than 20 over a 50-bp sliding window were then truncated, using Trimmomatic (Version 0.33).

### 2.3. Characterization of the Microbial Community and Gene Functional Prediction

The sequences were clustered and classified into operational taxonomic units (OTUs) at a 97% sequence similarity threshold with a Venn diagram using Usearch (Version 1.1.0) software (http://picrust.github.io/picrust/, (accessed on 27 October 2021)). The OTU abundance was standardized for further analyses. Microbial community richness and diversity were analyzed using alpha diversity estimators, including the Ace, Chao 1, Simpson, and Shannon indices, which were calculated using Mothur (Version v.1.30.2, https://mothur.org/wiki/calculators/, (accessed on 27 October 2021)). Non-metric multidimensional scaling (NMDS) was used to evaluate the beta diversity with QIIME (http://qiime.org/scripts/assign_taxonomy.html, (accessed on 27 October 2021)). The taxonomy was determined using the RDP Classifier (Version 2.11, http://sourceforge.net/projects/rdp-classifier/, (accessed on 27 October 2021)), with a confidence threshold of 70% at the phylum and genus levels [21]. A Spearman correlation and cluster analysis were performed to show the relationships among the elements and microbes [22]. A Kruskal–Wallis H-test was performed to assess the significant differences in taxa abundance. Molecular ecological networks (MENs) were constructed using the Networkx software (Los Alamos National Laboratory, Los Alamos, NM, USA) to determine the coexistence of taxa among the samples [23]. The clusters of orthologous groups (COG) data were obtained and computed using the Greengenes ID for gene function prediction with PICRUSt software, Version 1.1.0 (http://picrust.github.io/picrust/, (accessed on 27 October 2021)) [24].

### 2.4. Chemical Analysis

The amounts of ammonia nitrogen (NH_4_^+^-N), TN, and TP, as well as the pH, influence microbial growth, while heavy metals increase the potential risk of disease or harm to humans and the environment. The NH_4_^+^-N, TN, TP, pH, zinc (Zn), iron (Fe), copper (Cu), manganese (Mn), cadmium (Cd), lead (Pb), and nickel (Ni) of the WWTPs were analyzed according to the corresponding standard methods [25]. Nessler’s reagent colorimetric method was used to determine the concentration of NH_4_^+^-N; the alkaline potassium persulfate elimination UV spectrophotometric method was used for the determination of TN concentration [26], and ammonium molybdate spectrophotometry was used to determine that of TP. The NH_4_^+^-N, TN, and TP were measured by a UV-2800 spectrophotometer (UNICU Analytical, Shanghai, China). The pH was determined using a Precision pH meter BPP-7800 (Bell Analytical, Hong Kong, China). The concentrations of HMs (Zn, Fe, Cu, Mn, Cd, Pb, and Ni) were estimated using a flame atomic absorption spectrophotometer AA-6300 (Shimadzu, Kyoto, Japan). All the treatments were performed in triplicate.

## 3. Results and Discussion

### 3.1. Sample Characteristics

The corresponding flow processes of the two WWTPs are shown in Figure S1 of the Supplementary Materials. The composition and concentration of the samples of the tested WWTPs are depicted in Table S1 of the Supplementary Materials. The concentrations of the HMs Zn, Fe, Cu, Mn, Cd, Pb, and Ni in the treatment units of both WWTPs were lower than the discharge standards (GB 18918-2002) [9]. It should be noted that the concentrations of Mn and Cd were very close to the upper limit. The concentrations of Mn and Cd were significantly higher in the units of the SM-WWTP than in those of the XW-WWTP. In contrast, the concentrations of Fe and Pb were lower in the units of the XW-WWTP than in those of the SM-WWTP. The concentrations of Mn, Fe, and Cu in the SM_O were significantly higher than in the SM_A, whereas the concentrations of Zn and Cd were the opposite. The concentrations of Fe and Zn were significantly higher in the XW_O than in the XW_M. Previous studies have also shown that HMs could affect the microbial community’s structure and alter the microbial species [27]. The presence of variation in HMs could also cause differences in the microbial community between different treatment units.

### 3.2. Microbial Community Diversity

In total, 1,213,808 valid DNA sequences were obtained. The coverage values for all samples were greater than 0.982, indicating that the corresponding sequence library provided complete coverage of the microbial communities. All the sequences were compared and classified into 5205 taxonomic operational units (OTUs), which were considered as possibly being close to the genus.

The microbial community’s richness and diversity indices of all the sludge samples are shown in Table 2. Generally, the XW-WWTP showed lower values for the Ace and Chao 1 indices, suggesting that microbial community richness here was lower than that in the SM-WWTP. The XW-WWTP had a larger standard deviation than that of the SM-WWTP, indicating that the microbial community richness in XW-WWTP was less stable than that in the SM-WWTP. It is hypothesized that the microbial richness was affected by the HMs in XW-WWTP. The XW-WWTP had a lower Shannon index and a higher Simpson index, implying that microbial community diversity was also lower than that in the SM-WWTP. In XW-WWTP, the highest Shannon index but the lowest Simpson index were present in XW_M, which indicated that the MBR unit had more microbial diversity than the other two units [28]. Moreover, in the SM-WWTP, the Shannon, Ace, and Chao 1 indices of SM_A were lower than those of the SM_O and Simpson index, inversely, denoting that both the microbial community richness and diversity of the SM_A were lower than those of the SM_O. Similar differences in microbial richness and diversity were observed in the activated sludge samples from all the other WWTPs. Previous studies on WWTPs found that industrial wastewater considerably reduced the numbers of bacteria, while municipal wastewater enhanced the microbial diversity of the sludge [29]. These findings were consistent with our results.

**Figure 1:**
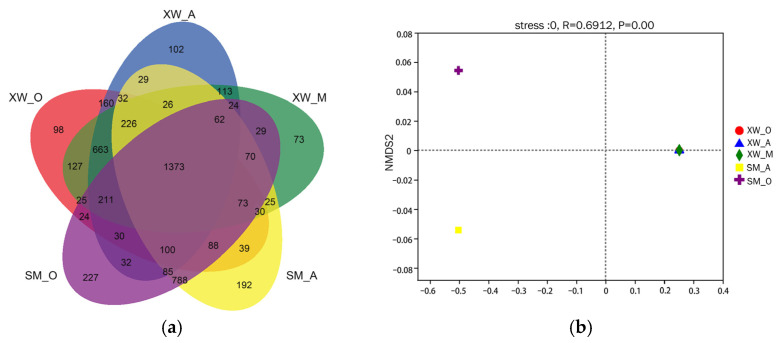
The similarity of the microbial community composition found in the wastewater treatment plants. (**a**) Venn diagram of the OTU distribution; (**b**) NMDS of the microbial community dynamics.

The Venn diagram reflected the microbial similarity and dissimilarity (Figure 1a) [21]. The degree of overlap was greater between the SM_A and SM_O than in the case of the XW_A, XW_M, and XW_O. As indicated in Figure 1, the average number of microbial OTUs in each unit of the XW-WWTP was lower than that of the SM-WWTP. This was attributed to the higher concentrations of HMs (specifically, Pb, Fe, and Cu) in the XW-WWTP, which were higher than those in the SM-WWTP, affecting the microbial variety [29]. This was consistent with previous findings that higher concentrations of HMs resulted in lower microbial richness [30]. The similarity of each sample was also indicated using nonmetric multidimensional scaling (NMDS) (Figure 1b). The XW A, XW M, and XW O were overlapping, suggesting that their microbial communities were similar. The SM A and SM O, on the other hand, were far apart, indicating that their microbial community structures differed.

### 3.3. Microbial Community Composition

**Figure 2:**
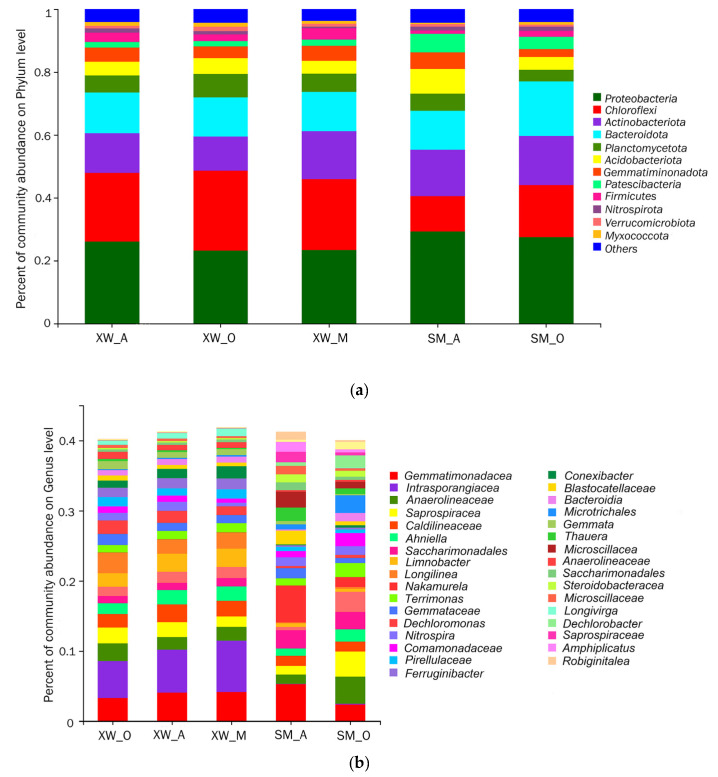
Microbial communities of the wastewater treatment plants: (**a**) phylum level; (**b**) genus level.

The taxonomic composition of each sample was evaluated at both the phylum (Figure 2a) and genus (Figure 2b) levels. The five most abundant phyla in all the samples were *Proteobacteria* (23.23–29.35%), *Chloroflexi*, *Actinobacteriota*, *Bacteroidetes* (12.40–17.34%), and *Planctomycetes*, in the order of their relative proportions. *Proteobacteria* and *Bacteroidetes* were found to be dominant in the WWTPs in previous studies [31], where they played a significant and extensive role in the removal of organic matter and nutrients. *Actinobacteria* and *Bacteroidetes* may play a key role in nitrogen transformation [32]. *Chloroflexi* was the most important bacterial phylum for nitrite oxidation and denitrification in activated sludge [33], playing a role in the production of the filamentous scaffolding of flocs, carbohydrate breakdown, and sludge settleability [34,35]. In addition, *Proteobacteria*, *Bacteroidetes*, *Acidobacteria*, *Firmicutes*, and *Nitrospirae* were the dominant phyla at different stages of the two WWTP projects in Shenzhen, China [36], with *Proteobacteria* being the most abundant, which was consistent with the findings of this experiment. The differences in other dominant phyla were likely due to the different quality of the influent samples, and the inhibition of the growth of microorganisms by the HMs contained in the industrial wastewater that was used in this experiment.

Typically, the most mutually abundant genera in all the samples were *Gemmatimonadaceae* (from 2.35 to 5.26%), *Anaerolineaceae* (from 1.32 to 3.91%), *Saprospiraceae* (from 1.22 to 3.61%), *Caldilineaceae* (from 1.41 to 2.5%), *Ahniella* (from 1.05 to 2.09%), *Terrimonas* (from 1.03 to 2.01%), and *Gemmataceae* (from 0.72 to 1.6%). Previous studies found that *Gemmatimonadaceae*, a Gram-negative bacterium, played a key role in nitrogen transformation [37]. *Anaerolineaceae* and *Caldilineaceae* belong to the *Chloroflexi* phylum and play important roles in denitrification. Previous reports showed that the dominant *Anaerolineaceae* ensured the effective treatment of traditional Chinese medicine (TCM) wastewater [38], while *Caldilineaceae* exhibited a high removal efficiency for COD (75.59%), TN (76.15%), and NH_4_^+^-N (83.23%) in biofuel cells (MFC) [39]. Therefore, these dominant bacteria were thought to serve as the functional microbes for the removal of organic matter, N, and P in the XW- and SM-WWTPs. Compared with the activated sludge of other A^2^O and MBR process WWTPs, their dominant bacterial genera were *Haliangium*, *Arcobacter*, *Ferruginibacter*, and *Cloacibacter* [36], which was very different from our results. The main reasons for this discrepancy could be differences in the locations of the WWTPs and the quality of the influent.

In addition, the XW-WWTP had the highest abundance of *Intrasporangiaceae*, varying from 5.25 to 7.3%, while the SM-WWTP had a much lower abundance that ranged between 0.06 and 0.09%. In contrast, the XW-WWTP exhibited a distinct SBR biofilm with Hg in the samples, but the SM-WWTP did not, which may explain the significant difference in *Intrasporangiaceae* content. Previous studies found that *Intrasporangiaceae* had high resistance to and high removal ability of Hg, as well as being a dominant genus with a high removal efficiency of N and P in wastewater treatment [40].

### 3.4. Effect of Environmental Factors on Microbial Community Structure

**Figure 3:**
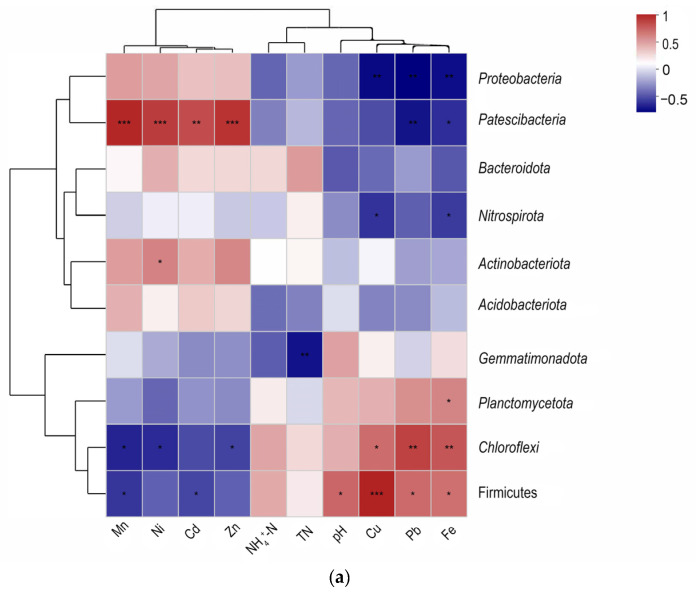

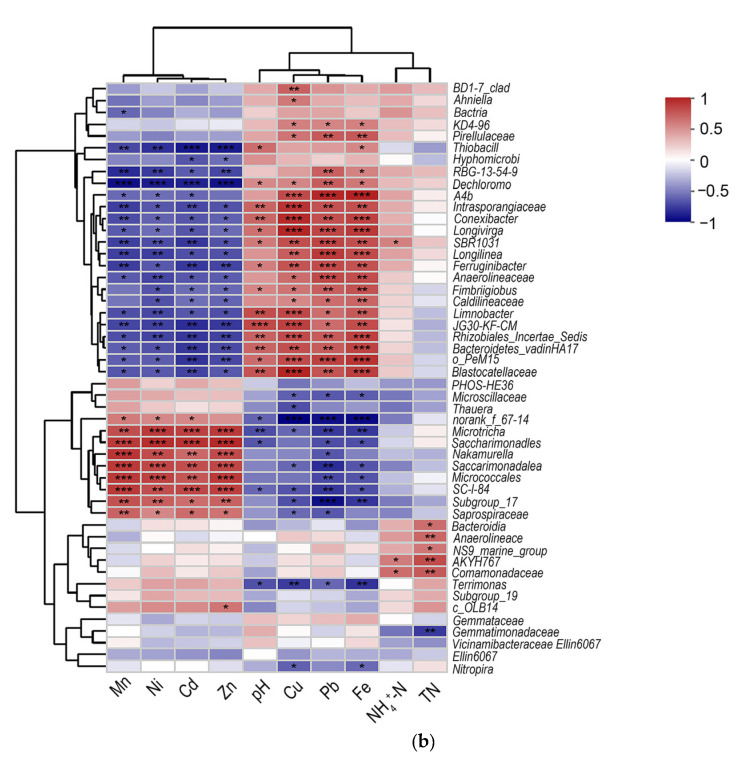
Spearman correlation heatmap of the studied environmental factors, showing the abundance of the dominant bacterial communities in the wastewater treatment plants: (**a**) phylum level; (**b**) genus level. Note: * 0.01 < *p* < 0.05; ** 0.001 < *p* < 0.01; *** *p* < 0.001.

Spearman’s correlation analysis was used to reveal the relationship between the microbial communities and the environmental factors at the phylum (Figure 3a) and genus (Figure 3b) levels. Previous reports revealed that N, P, and pH were the most important factors affecting the microbial communities in wastewater treatment [41].

In this study, pH, TN, and NH_4_^+^-N were selected as the environmental factors. The results showed that the pH had a significant positive impact on the phylum *Firmicutes*. TN displayed a strong negative correlation with the phylum *Gemmatimonadota*. The HMs of Mn, Ni, Cd, and Zn displayed a strong positive correlation with the phylum *Patescibacteria*, but a strong negative correlation with the phyla *Chloroflexi* and *Firmicutes*. The HMs of Cu, Pb, and Fe presented a strong positive correlation with the phyla *Chloroflexi* and *Firmicutes* but were significantly negatively correlated with *Proteobacteria*. Earlier reports showed that Cd, Pb, and/or Zn were significantly positively correlated with the abundances of *Chloroflexi* [42], *Firmicutes* [43], and *Patescibacteria* [44], but significantly negatively correlated with the abundance of the *Proteobacteria* in wastewater treatment. Our findings are similar to these results.

The pH had a significant positive impact on the bacterial genera of *lntrasporangiaceae*, *Conexibacter*, *Limnobacter*, *Bacteroidetes*, and *Blastocatellaceae* (*p* < 0.01), but had a significant negative effect on *Microtrichales* and *Saccharimonadales* (*p* < 0.05 or 0.01). Previous reports showed that pH was strongly positively correlated with the abundance of *Conexibacter* [45,46], *Limnobacter* [47], and *Bacteroidetes* [48], while pH was strongly negatively correlated with *Saccharimonadales*. Similarly, TN displayed a significant positive correlation with the genera *Comamonadaceae* and *Anaerolineaceae*. NH_4_^+^-N displayed a significant positive correlation with the genus *Comamonadaceae*. *Comamonadaceae* have been reported as being denitrifying and phosphorus-polymerizing bacteria [49]. *Anaerolineaceae* have been found to be widespread in anaerobic reactors, degrading sugars and some cellular tissues [50]. These findings are in accordance with our results.

HMs had a greater impact on the dominant bacterial genera, compared to the water quality indicators of NH_4_^+^-N, TN, and pH. A significant positive correlation was found between the HMs Mn, Ni, Cd, and Zn, and the genera of *Saccharimonadales*, *Nakamurella*, *Micrococcales*, and *Microtrichales*; however, a significant negative correlation was found between the same HMs and *Thiobacillus* and *Dechloromonas*. The HMs Cu, Pb, and Fe showed a significant positive correlation with the genera *Longilinea* and *Ferruginibacter* but had a significant negative correlation with *Micrococcales*. The proportion of *Saccharimonadales* increased significantly in the SM_A, with slightly high Zn, probably due to their strong ability to degrade complex organic matter in the environment of HMs, which is in agreement with prior studies [51,52]. *Micrococcales* showed significant alterations under high concentrations of Cu and/or Pb [53]. *Thiobacillus* and Dechloromonas decreased their activity in landfill leachate that contained high concentrations of Cd [54]. Based on previous reports and the present results, the bacterial genera *Saccharimonadales*, *Nakamurella*, *Micrococcales*, and *Microtrichales* have the potential to be resistant to Mn, Ni, Cd, and Zn. These bacteria could be employed for the adsorption of HMs in wastewater treatment, as they showed potential HMs resistance capacity. The present study may help to explore the microbial community in an environment that contains HMs, which is of benefit to environmental safety when learning how to mitigate HM pollution.

In addition, some characteristic factors (nitrite, nitrate, and COD) could also affect the bacterial community [36]. TN and pH were studied in concert with the NH_4_^+^-N, and they played an important role in the basic activities of microorganisms. As shown in Figure 3a,b, bacteria that strongly correlated with NH_4_^+^-N, TN and HMs occupied an important position in the biological treatment of WWTPs and indirectly reduced the risk of hazards to human health and the environment by reducing the discharge of nitrogen, phosphorus, and HMs. Previous studies found that biochar enhanced the immediate promotion of anaerobic digestion performance, as well as nitrogen and phosphorus removal [55]. Based on these results, the addition of biochar might also be utilized to treat chemical wastewater, which could facilitate the removal of HMs [56]. Moreover, researchers should also consider the potential life-cycle impacts of wastewater that contains chemicals. The concentrated sludge that contains HMs should also be treated properly to decrease its risk to the environment and to human health [57].

### 3.5. Differentiation of the Microbial Communities

**Figure 4:**
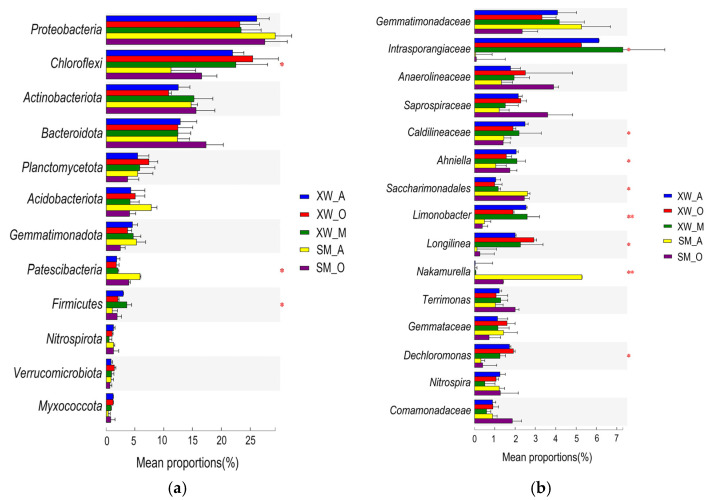
The relative abundance variation of top bacteria in the samples of WWTPs: (**a**) phylum level; (**b**) genus level. Note: * 0.01 < *p* < 0.05; ** 0.001 < *p* < 0.01.

The Kruskal–Wallis H test was used to assess the abundance differences among the 12 most abundant bacterial phyla (Figure 4a). The abundances of the bacterial phyla *Proteobacteria*, *Actinobacteriota*, and *Bacteroidota* were high in all five reaction tanks, without significant differences (*p* > 0.05). Other bacterial phyla, such as *Chloroflexi*, *Patescibacteria*, and *Firmicutes* had significant abundance differences (*p* < 0.05). The abundance of *Chloroflexi* was significantly higher in the three units of XW-WWTP than in the two units of SM-WWTP, while it was the opposite for the abundance of *Patescibacteria*. According to prior microbial community composition studies that were conducted at the phylum level, the majority of bacteria belonging to *Proteobacteria* have nitrification and denitrification functions. *Patescibacteria*-centric coexisting bacteria were essential for the anammox ecosystem because they produced anammox substrates and scavenged organic compounds in the anammox reactor [58]. Bacteria belonging to *Chloroflexi* were more tolerant to HMs [42] and accounted for the significantly higher abundance in the three units of XW-WWTP than in the two units of SM-WWTP. It was assumed that the AO-MBR process of XW-WWTP was more effective than the AO process of SM-WWTP in removing HMs. The rest of the dominant phyla showed some stability under different processes. Some dominant phyla were more sensitive to the changes in sewage processes, and their abundance changed significantly as the sewage processes were altered.

The Kruskal–Wallis H test was also used to evaluate the abundance differences of the 15 most abundant bacterial genera (Figure 4b). The abundances of the bacterial genera *Gemmatimonadaceae*, *Anaerolineaceae*, *Saprospiraceae*, and *Terrimonas* showed no significant differences (*p* > 0.05) among the five reaction tanks. Other bacterial genera, such as *Intrasporangiaceae*, *Caldilineaceae*, *Ahniella*, *Saccharimonadales*, *Longilinea,* and *Dechloromonas* displayed significant abundance differences (0.01 < *p* < 0.05). The abundances of *Limnobacter* and *Nakamurella* were significantly different (*p* < 0.01). As a result, these bacterial genera contributed to the variances in the microbial communities among the five reaction tanks. The abundances of *Intrasporangiaceae* and *Limnobacter* were significantly higher in the AO-MBR process of XW-WWTP than in the AO process of SM-WWTP. When combined with the previous analyses of microbial community composition, *Intrasporangiaceae* and *Limnobacter* differed considerably, probably due to the different types and concentrations of HMs and environmental factors in the WWTPs [40,59].

### 3.6. Microbial Ecological Network Analysis

**Figure 5:**
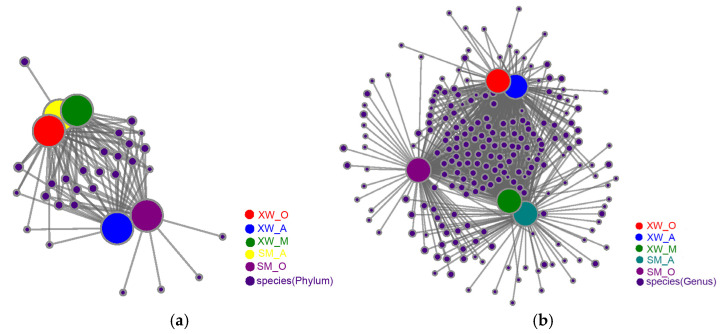
MEN of the microbial populations (**a**) phylum level; (**b**) genus level.

Since activated sludge is a group of microorganisms with multiple functions, microbial interactions have an important influence on the formation of functional groups and the removal of pollutants. Therefore, the symbiotic relationships among these dominant species and their ecological functions have been explored. Figure 5 illustrates the molecular ecological network (MEN) populations at the phylum and genus levels.

Table 3 shows that the bacterial phyla *Proteobacteria*, *Chloroflexi*, *Actinobacteriota*, *Bacteroidota*, and *Planctomycetota* had the five highest weighted degrees, varying from 52,766 to 11,367, whereas the bacterial genera *Gemmatimonadaceae, Intrasporangiaceae, Anaerolineaceae, Saprospiraceae, Caldilineaceae, Ahniella, Saccharimonadales, Limnobacter, Longilinea*, and *Nakamurella* had the ten highest weighted degrees of 7810 to 2779. These genera are all members of the phyla mentioned above. They showed a high correlation with the HMs, TN, and NH_4_^+^-N, demonstrating their function in nitrogen removal and the adsorption of HMs (Figure 3). *Anaerolineaceae*, which are considered to play an important role in the degradation of organic pollutants [60], exhibited the strongest positive relationship with TN. *Proteobacteria* was the dominant genera in terms of anaerobic granular sludge and anaerobic digestion for methane production, whereas *Chloroflexi*, *Actinobacteriota*, and *Bacteroidota* were less abundant in low-carbon sources containing Se and Cd [61]. In general, the correlation network analysis revealed that the main functional bacteria aggregated together, based on their unique metabolic characteristics, and collaborated to play a remarkable role in wastewater treatment [52].

There were many more links present at the genus level than at the phylum level (Table 4). Node connectivity at the phylum level was XW_A (56), SM_O (55), XW_O (55), XW_M (53), and SM_A (52). Node connectivity at the genus level was XW_A (904), XW_O (889), SM_O (872), SM_A (868), and XW_M (855). Overall, the number of nodes of the XW-WWTP was greater than that of the SM-WWTP at both the phylum and genus levels, indicating that the MEN of the XW-WWTP was more complex, with a more stable microbial community structure for the treatment of wastewater containing HMs [62]. The dominant genera played a more significant role in the XW_A than in the XW_M because XW_A had a higher number of nodes than XW_M at both the phylum and genus levels. The results at the phylum level differed slightly from those at the genus level because these phyla and genera played different roles in the activated sludge of the different treatment units. In general, the MEN analysis indicated that the main functional microbes in the two WWTPs were clustered based on their distinct metabolic characteristics and played remarkable roles in collaborative pollutant removal [52].

### 3.7. Microbial Functional Prediction

**Figure 6:**
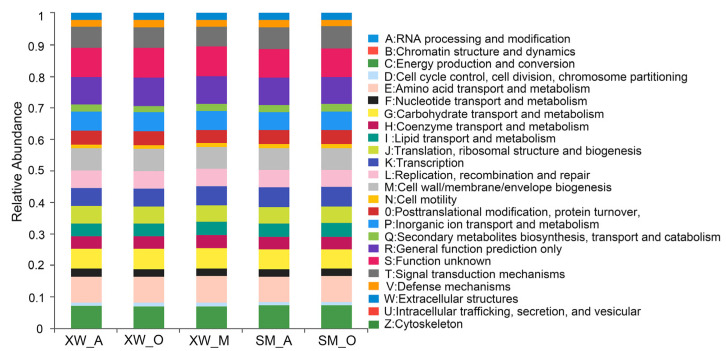
The functional gene prediction of the sludge samples, annotated by the COG database.

The metabolic activities of microorganisms in WWTPs played a crucial role in the overall operational performance of pollutant removal processes [52]. Bacterial functional prediction based on COG-level functional categories is illustrated in Figure 6. In general, the relative abundance of general function prediction was 8.85%, followed by amino acid transport and metabolism (8.33%), transcription (8.33%), energy production and conversion (7.10%), cell wall/membrane/envelope biogenesis (6.99%), carbohydrate transport and metabolism (6.27%), and inorganic ion transport and metabolism (5.90%). Previous studies reported high proportions of the genes responsible for these functions in biological reactors [63]. The higher the relative abundance of COG functional categories, the greater the role they play in cell growth and reproduction. It was apparent that the most abundant genes in each sample were the related metabolic genes.

In the SM_O and SM_A tanks of the SM-WWTP, the relative abundance of amino acid transport and metabolism and general function prediction exceeded 8%, whereas the relative abundance of RNA processing and modification, the chromatin structure and dynamics, extracellular structures, and the cytoskeleton was below 1%. In the three units of XW-WWTP, the relative abundance of amino acid transport and metabolism general function prediction was high. These functional proteins were shown to be closely related to the life activities of the microorganisms [64], suggesting that microbial communities have a key function in removing various pollutants. However, the proportions of RNA processing and modification, chromatin structure and dynamics, extracellular structures, and the cytoskeleton were also low. By determining the relationship between these genes and microbial metabolic activities, it is possible to maximize the role of HM removal to protect the water environment from HM pollution.

## 4. Conclusions

Microbial community analysis showed that both microbial richness and diversity were lower in the XW-WWTP than in the SM-WWTP. The dominant phyla and genera in the two WWTPs were similar, except that the proportions of *Intrasporangiaceae* (5.25~7.3%) in the XW-WWTP were much higher than those in the SM-WWTP (0.06~0.09%). In particular, the abundance of *Chloroflexi* (21.93~25.45%) in the XW-WWTP was significantly greater than that in the SM-WWTP (11.24~16.56%). The results offer a comprehensive outlook for microbial community characterization and for optimizing the wastewater treatment processes in WWTPs located in industrial zones, which could reduce the risks of effluent from WWTPs that threaten human health and environmental safety.

## Figures and Tables

**Table 1:** Designed influent quality of raw sewage in the wastewater treatment plants examined (unit: mg/L).

	BOD_5_	COD	NH_3_-N	TN	TP
SM-WWTP	130	300	25	35	4
XW-WWTP	130	250	20	35	3

BOD_5_: Biochemical oxygen demand; COD: chemical oxygen demand; NH_3_-N: nitrate nitrogen; TN: total nitrogen; TP: total phosphorus.

**Table 2:** Alpha diversity indices (bacterial richness and diversity) of the microbial communities found in the wastewater treatment plants.

Sample	Shannon	Simpson	Ace	Chao	Coverage
SM_A	5.963 ± 0.079	0.009 ± 0.001	3106.841 ± 44.726	3070.979 ± 65.839	0.982 ± 0
SM_O	6.247 ± 0.106	0.005 ± 0.001	3148.173 ± 98.253	3170.752 ± 86.135	0.982 ± 0
XW_A	5.906 ± 0.125	0.01 ± 0.001	2920.2 ± 143.276	2945.665 ± 152.135	0.983 ± 0.001
XW_M	5.873 ± 0.054	0.01 ± 0.001	3026.602 ± 284.754	2963.67 ± 71.806	0.983 ± 0.001
XW_O	5.957 ± 0.06	0.009 ± 0.002	2973.613 ± 126.663	3003.2 ± 164.09	0.983 ± 0.001

SM: SM-WTTP; XW: XW-WTTP; A: anaerobic tank; O: aerobic tank; M: membrane bioreactor.

**Table 3:** The bacterial nodes at the phylum and genus levels of the microbial communities associated with the wastewater treatment plants.

	Node Name (The Top)	Degree	Weighted Degree
Phylum	*Proteobacteria*	5	52,766
	*Chloroflexi*	5	39,755
	*Actinobacteriota*	5	28,079
	*Bacteroidota*	5	27,485
	*Planctomycetota*	5	11,367
Genus	*Gemmatimonadaceae*	5	7810
	*Intrasporangiaceae*	5	7659
	*Anaerolineaceae*	5	4662
	*Saprospiraceae*	5	4394
	*Caldilineaceae*	5	3840
	*Ahniella*	5	3472
	*Saccharimonadales*	5	3371
	*Limnobacter*	5	3218
	*Longilinea*	5	3092
	*Nakamurella*	5	2779

**Table 4:** The samples nodes at the phylum level and genus levels of the microbial communities associated with the wastewater treatment plants.

Phylum Level		Genus Level	
Node Name	Degree	Node Name	Degree
SM_A	52	SM_A	868
SM_O	55	SM_O	872
XW_A	56	XW_A	904
XW_O	55	XW_O	889
XW_M	53	XW_M	855

SM: SM-WTTP; XW: XW-WTTP; A: anaerobic tank; O: aerobic tank; M: membrane bioreactor.

## Data Availability

The data presented in this study are available in the manuscript or Supplementary Materials.

## Supplementary Materials

The following supporting information can be downloaded at: https://www.mdpi.com/article/10.3390/ijerph19116529/s1. Figure S1: Process flow of the wastewater treatment plants; Table S1: Composition and concentrations of wastewater samples in the tested wastewater treatment plants (unit: mg/L).

## Abbreviations

WWTP	Wastewater treatment plant
HM	Heavy metal
AS	Activated sludge
SS	Suspended solid
TP	Total phosphorus
NH_3_-N	Nitrate nitrogen
TN	Total nitrogen
COD	Chemical oxygen demand
BOD_5_	Biochemical oxygen demand
MBR	Membrane bioreactor
SRT	Sludge retention time
