# Supplementary material for: Characterization of Microbial Communities in Wastewater Treatment Plants Containing Heavy Metals Located in Chemical Industrial Zones
Source: Int J Environ Res Public Health. 2022 May 27;19(11):6529. doi: 10.3390/ijerph19116529 (PMC9180875; doi:10.3390/ijerph19116529)
Supplement: Supplementary file 1 [file ijerph-19-06529-s001.zip › ijerph-1706994-supplementary.pdf]

Figure S1 Process flow of the wastewater treatment plants

Table S1. Composition and concentrations of wastewater samples in the tested wastewater treatment plants (unit: mg/L).

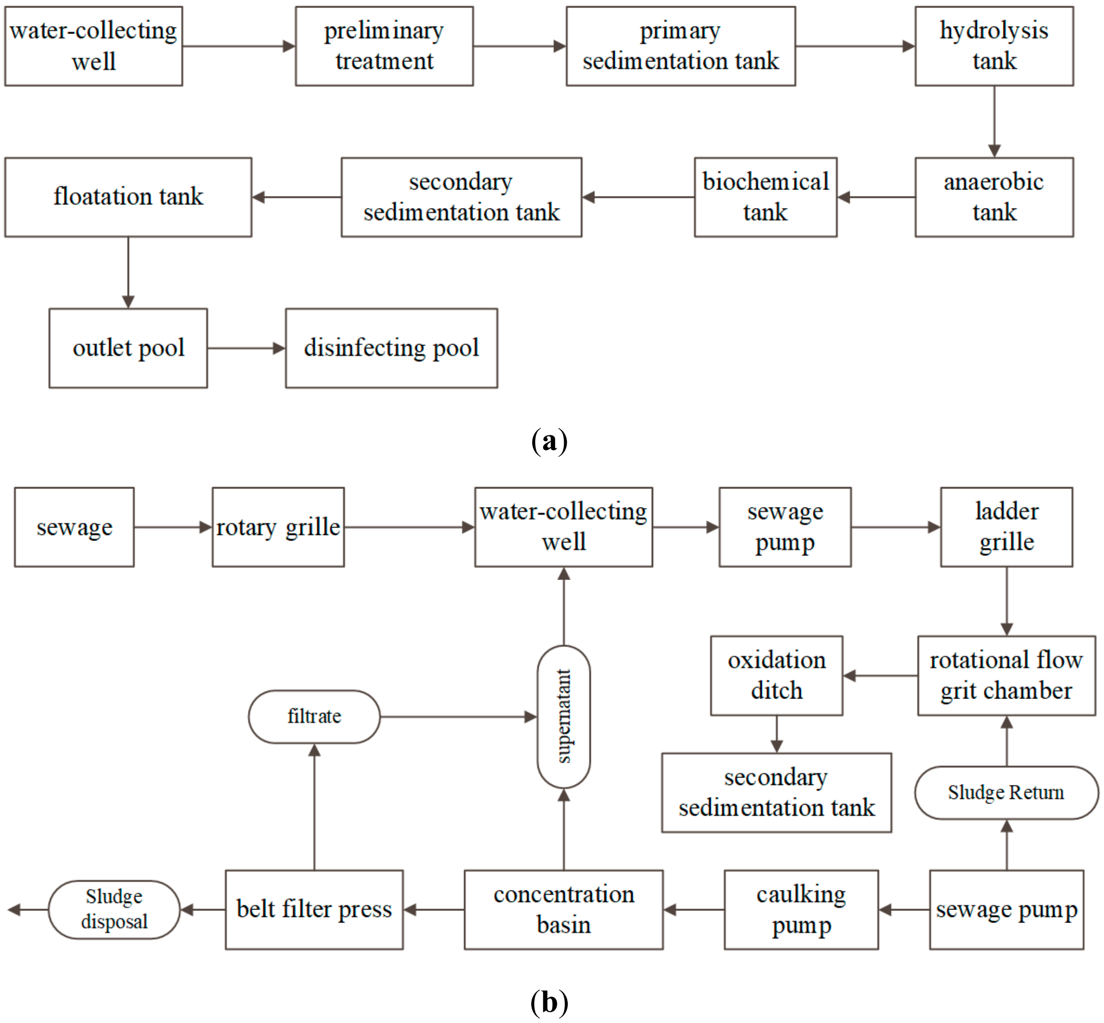

Figure. S1 Process flow of the wastewater treatment plants. (**a**) SM-WWTP process flow; (**b**) XW-WWTP process flow.

Table S1. Composition and concentrations of wastewater samples in the tested wastewater treatment plants (unit: mg/L).

samples	pH	NH_4^+-N	TN	Mn	Fe	Ni	Cu	Zn	Cd	Pb
SM_A	7.04	2.571	4.875	1.226	0.020	0.023	0.012	0.371	0.008	0.003
SM_O	6.68	5.474	9.561	1.798	0.097	0.021	0.021	0.231	0.004	0.002
XW_A	7.22	4.593	6.394	0.018	0.368	0.003	0.026	0.095	0.000	0.024
XW_M	7.12	5.163	6.210	0.027	1.827	0.004	0.030	0.159	0.000	0.075
XW_O	7.03	4.956	6.813	0.025	1.548	0.003	0.021	0.144	0.000	0.074

SM: SM-WTTP; XW: XW-WTTP; A: Anaerobic tank; O: Aerobic tank;

M: membrane bioreactor
